# Review and Meta-Analyses of the Effects of MLC601/MLC901 (NeuroAiD) on Post-Stroke Functional and Motor Recovery

**DOI:** 10.3390/neurolint18080141

**Published:** 2026-07-23

**Authors:** Narayanaswamy Venketasubramanian, Tsong-Hai Lee, Hou Chang Chiu, Liang Guo, Christopher Li Hsian Chen

**Affiliations:** 1 Raffles Neuroscience Centre, 585 North Bridge Road, #09-00 Raffles Hospital, Singapore 188770, Singapore; 2 Memory Aging and Cognition Centre, Department of Pharmacology, Yong Loo Lin School of Medicine, National University of Singapore, Blk MD3, 16 Medical Drive, #04-01, Singapore 117600, Singapore; cplhchen@yahoo.com.sg; 3 Linkou Chang Gung Memorial Hospital, College of Medicine, Chang Gung University, Taoyuan City 333, Taiwan; thlee@cgmh.org.tw; 4 Shuang Ho Hospital, Taipei Medical University, Taipei 23561, Taiwan; m001012.hc@gmail.com; 5 Singapore Clinical Research Institute, Consortium for Clinical Research and Innovation, Singapore 139234, Singapore; liang.guo@scri.cris.sg

**Keywords:** MLC901/MLC601, NeuroAiD, functional recovery, motor recovery, ischaemic stroke, meta-analysis, systematic review

## Abstract

**Background:** Post-stroke recovery varies widely, and pharmacological options to enhance rehabilitation outcomes remain limited. MLC601/MLC901 (NeuroAiD), a natural neurorestorative product, has been evaluated as an adjunct to standard care to improve functional and motor recovery after ischaemic stroke. This systematic review and meta-analysis assessed its efficacy using validated outcome measures. **Methods**: A systematic PubMed search identified randomised controlled trials comparing MLC601/MLC901 with placebo or active comparators in adults with ischaemic stroke. Functional outcomes included modified Rankin Scale (mRS), Barthel Index (BI), and Diagnostic Therapeutic Effects of Apoplexy (DTER) item 8 scores. Motor outcomes included Fugl–Meyer Assessment (FMA), DTER motor items, and National Institutes of Health Stroke Scale (NIHSS) motor scores. Data were pooled using fixed- and random-effects models. Odds ratios (ORs) and standardised mean differences (SMDs) were calculated. Risk of bias was assessed using the Cochrane RoB 1.0 tool. **Results**: Six publications reporting seven randomised clinical studies were included in the meta-analysis. Of the 7 studies, 5 were assessed as having a low risk of bias, while 2 were assessed as having an unclear risk. Altogether, 1535 participants for functional outcomes and 1774 for motor outcomes were analysed. Functional recovery significantly favoured MLC601/MLC901 at 1 month (OR 2.61; *p* = 0.004), 6 months (OR 1.38; *p* = 0.002), 12 months (OR 1.33; *p* = 0.03), and end-of-study (OR 1.40; *p* = 0.007), with benefits persisting up to 24 months. Motor recovery was assessed at months 1, 2 and 3 and at the end of the study. It also improved consistently over time, with the greatest effects during the first two months. Benefits were most evident in patients with moderately severe stroke (NIHSS 8–14). Clinical studies consistently indicate that NeuroAiD is safe and well-tolerated as an adjunct to standard ischaemic stroke care. **Conclusions:** MLC601/MLC901 is associated with improved functional independence and motor recovery after ischaemic stroke. Benefits appear within 1 month and may persist for up to 2 years, supporting early use alongside rehabilitation to optimise recovery.

## 1. Introduction

Stroke remains one of the leading causes of death and long-term disability worldwide. Globally, more than 12 million people experience a new stroke each year, and over 100 million individuals live with its consequences, resulting in substantial personal, social, and economic burden [1].

Despite advances in acute stroke management, long-term recovery remains suboptimal for many patients. Stroke unit care, early rehabilitation, and evidence-based acute interventions such as intravenous thrombolysis (IV rtPA) and endovascular thrombectomy have improved survival and early clinical outcomes [2]. However, their proven benefits have primarily been shown in settings with highly organised and specialised neurovascular interventional services. These treatments are time-dependent, applicable to only a subset of patients, and not universally accessible, particularly in low- and middle-income countries (LMICs) [3]. As a result, many stroke survivors experience persistent disability and require prolonged rehabilitation.

The impact of stroke is particularly pronounced in Asia, where nearly two-thirds of the world’s stroke deaths occur and where incidence rates have continued to rise due to population ageing, urbanisation, and the increasing prevalence of vascular risk factors [4]. Consequently, stroke represents a major public health challenge in many Asian countries, with significant implications for healthcare systems and rehabilitation services.

Recovery outcomes are strongly influenced by baseline stroke severity [5]. Patients with moderate to severe strokes, as commonly defined by a National Institutes of Health Stroke Scale (NIHSS) score of 8 to 14 or higher, have substantially lower rates of functional independence than those with mild strokes. In large clinical datasets, patients with mild deficits frequently recover spontaneously, whereas those with moderate neurological impairment have a significantly lower probability of achieving favourable outcomes such as a modified Rankin Scale (mRS) score of 0–1 [6]. Thus, in the CHIMES study [7] conducted in patients who had suffered an ischaemic stroke of intermediate severity within 72 h and were treated with MLC601 and monitored for 3 months, approximately two-thirds of patients with mild stroke achieved favourable outcomes at three months, compared with only about one-third of those with baseline NIHSS scores of 8–14 [8]. This latter subgroup, therefore, represents a clinically important population with significant unmet therapeutic needs.

Although rehabilitation therapies remain the cornerstone of post-stroke management, pharmacological options that enhance neurological recovery are limited. Numerous candidate neuroprotective and neurorestorative agents have been investigated over the past decades, but most have failed to demonstrate consistent clinical benefit in randomised trials [9]. Consequently, current stroke guidelines place a strong emphasis on acute reperfusion therapies and secondary prevention strategies, with few medications recommended specifically to promote neurological recovery [10]. This therapeutic gap emphasises the need for interventions that can enhance neuroplasticity, support neural repair, and improve functional recovery when used alongside standard rehabilitation.

The Asian Stroke Advisory Panel (ASAP) recently published a systematic review of treatments evaluated for their potential to enhance or promote recovery in adult stroke patients [11]. Eligible treatments were those that had obtained registration or marketing authorisation in at least one country, regardless of their original therapeutic indication. The review considered outcomes related to neurological impairments as well as measures of function and disability. From the available literature, studies providing the highest level of evidence were identified. They were assigned a level of evidence (LOE) by expert consensus, using the American Stroke Association’s LOE scheme [12]. MLC601/MLC901 (NeuroAiD) was granted an LOE of B-R (Randomised) for three out of four analysed studies, alongside six other products: edaravone and its combination with dexborneol (1 B-R each), cinepazide (1 B-R), clomethiazole (1 B-R), SSRIs (1 B-R for citalopram), cerebrolysin (1 B-R), and Panax notoginseng (1 B-R, but in intracranial haemorrhage, not in ischaemic stroke studies).

MLC601 and its simplified formulation MLC901 (NeuroAiD) are natural neurorestorative compounds derived from traditional Chinese medicine (TCM) and have been investigated for their potential to enhance recovery after ischaemic stroke. Preclinical studies suggest that these formulations exert multimodal effects that may promote neurological recovery, including neuroprotection, stimulation of neurogenesis and angiogenesis, enhancement of neuroplasticity and synaptic remodelling, modulation of inflammatory responses, and protection against excitotoxicity and oxidative stress. These complementary mechanisms are believed to facilitate endogenous brain repair and functional recovery after cerebral injury [13]. A comprehensive review of the pharmacological and clinical evidence supporting these mechanisms has recently been published [14].

The present study, therefore, conducted a comprehensive systematic review and meta-analysis of randomised controlled trials to evaluate the effects of MLC601 and MLC901 on post-stroke functional independence and motor recovery, using validated clinical outcome measures.

MLC901 is marketed under the trade name NeuroAiD II (or NurAiD II) in 39 countries across seven global regions (Africa: three, ASEAN: eight, Balkans: five, CIS: four, Europe: 12, Middle East: six, South Asia: one). It is a natural product made from extracts of nine herbal ingredients, including 0.800 g per capsule of *Radix astragali* and 0.160 g per capsule of each of the following: *Radix salviae miltiorrhizae*, *Radix paeoniae rubra*, *Rhizoma chuanxiong*, *Radix angelicae sinensis*, *Carthamus tinctorius*, *Prunus persica*, *Radix polygalae*, and *Rhizoma acori tatarinowii* [13]. It is a simplified version of its predecessor MLC601 (NeuroAiD), derived from TCM approved by the China Food and Drug Administration in 2001 for recovery after acute ischaemic stroke (AIS). MLC601 (NeuroAiD^TM^) and MLC901 (NeuroAiD^TM^II/NurAiD^TM^II) are two distinct proprietary formulae that have been shown to be equivalent in several pharmacological studies and are referred to as “Neuroaid” in this document. It is administered alongside standard stroke care, including control of vascular risk factors and rehabilitation as prescribed by investigators. We recently reviewed and summarised most of the pharmacological and clinical studies of Neuroaid for stroke in a recent publication [14].

In the present analysis, we conducted a comprehensive review and meta-analysis of the available evidence to assess the effects of Neuroaid on post-stroke functional and motor recovery. This research adds to previously published meta-analyses of the effect of MLC601/MLC901 on functional recovery [7,15], by adding additional trials with larger sample sizes, longer follow-up, as well as exploration of motor-recovery, and treatment effect in patients with moderately severe stroke (baseline NIHSS score of 8–14), a subgroup with great unmet rehabilitation needs.

Using the PICO framework, we evaluated whether MLC601/MLC901 (Intervention), compared with placebo or active comparator (Comparator), improves functional and motor recovery outcomes (Outcomes) in adults with ischaemic stroke (Population).

## 2. Methods

### 2.1. Objectives

The primary objective of this review and meta-analysis was to assess the effect of Neuroaid in addition to standard stroke care on patients’ functional and motor recovery after stroke. Specific aims included:

• Determining the effect of MLC601/MLC901 on long-term functional outcomes using validated global disability and independence measures.
• Determining its impact on motor function recovery using validated motor performance scales.
• Detecting any heterogeneity in the treatment effects in a subgroup of patients with moderately severe stroke (baseline NIHSS score 8–14), who are less likely to recover spontaneously.

### 2.2. Data Sources and Selection Criteria

The literature search was primarily based on PubMed and supplemented by proprietary publication records maintained by the NeuroAiD manufacturer and by manual cross-referencing. The PubMed search used combinations of the following terms: (“MLC601” OR “MLC901” OR “NeuroAiD-II” OR “NurAiD-II” OR “NeuroAid”) AND (“stroke” OR “ischaemic stroke” OR “ischemic stroke” OR “stroke recovery” OR “post-stroke rehabilitation”). Searches were conducted in titles, abstracts, and indexed terms when available. The search was limited to human studies and English-language publications. No geographic restrictions were applied. Then, we sorted studies by using key search terms such as MLC601 OR MLC901, NeuroAiD, stroke recovery, and post-stroke rehabilitation. Only randomised controlled trials (RCTs) were included. Studies were included if they reported standardised and validated outcome measures, such as (i) for functional outcomes, Diagnostic Therapeutic Effects of Apoplexy (DTER) item-8 scoring [16,17], Barthel Index (BI) [18], and mRS [7,19], and (ii) for motor outcomes, DTER motor items [16], Fugl–Meyer Assessment (FMA) motor score [20,21], and NIHSS motor scores [6,7,19].

The study population included adult patients with ischaemic stroke at various stages post-onset, enrolled in RCTs comparing MLC601/MLC901 with placebo on top of standard care. Most of the included studies were conducted in Asian countries and regions, with a few in the Middle East region. For the functional recovery analysis, studies covering a wide range of stroke severities were included. The selection ensured inclusion of trials with clearly defined endpoints, at least 3-month follow-up, and comparator arms receiving placebo alongside standard care.

Any preclinical studies, open-label efficacy papers, endpoints other than functional and motor outcomes, clinical safety papers, other subgroups, systematic reviews, and protocols were excluded.

As this research is a literature review with meta-analysis, rather than a full-scale systematic review, it is not eligible for registration in PROSPERO.

### 2.3. Data Extraction

For each included study, original data were retrieved and recorded using a standardised data collection form. The data extraction was performed by the author from the Singapore Clinical Research Institute (SCRI) and the Consortium for Clinical Research and Innovation (CRIS), Singapore. The data analysis was also conducted by this author and by the corresponding author. Extracted data included (i) study characteristics (author information, year of publication, country, study design, inclusion and exclusion criteria, and sample size), (ii) baseline characteristics (age, sex, stroke type, time from onset to treatment, and baseline NIHSS score), (iii) the treatment regimen (MLC601/MLC901 dosage and administration frequency), and (iv) the outcome measures for functional recovery, defined as DTER item 8 = 0, mRS 0–1, or BI ≥ 85, treated as binary outcomes, while motor recovery was treated as a continuous outcome across different scales, such as DTER motor items, FMA, or NIHSS motor items. Measurements were collected at multiple time points (1, 3, 6, 12, 18, 24 months, and at the end of the study), with motor assessments at 1, 2, and 3 months, and at the end of the study.

### 2.4. Statistical Analyses

All eligible studies were included in the meta-analysis whenever possible. To address heterogeneity across studies, data were combined using a random-effects model. However, the choice between fixed- and random-effects models depends on the situation. The random-effects model is usually preferred because it accounts for heterogeneity across studies [22]. However, when only a few studies are available, or when a large, high-quality study outweighs smaller, lower-quality ones, a fixed-effects model might be more suitable. For completeness, results from both random- and fixed-effects models are presented in the current analyses.

I^2^ (I-squared) quantifies the extent to which the variation between study results is due to real differences (heterogeneity) rather than chance. I^2^ values of 25%, 50%, and 75% indicate low, moderate, and substantial heterogeneity, respectively [23]. The summary effect for binary outcomes was reported as odds ratios (ORs) with 95% confidence intervals (CIs), and for continuous outcomes as standardised mean differences (SMDs) with 95% CIs. Whenever possible, sensitivity analyses using random-effects models were conducted to assess the robustness of the results.

A subgroup analysis was performed on patients with baseline NIHSS scores of 8–14 in the CHIMES (3-month follow-up) and CHIMES-E (6-month follow-up) studies [6]. The rationale for focusing on this subgroup after excluding patients with baseline NIHSS score 6–7 is that these patients with milder stroke severity tend to recover spontaneously, whereas there is a greater unmet need to improve neurorehabilitation for patients with more severe stroke (NIHSS 8–14).

The Mantel–Haenszel (M–H) method was used for dichotomous data and for repeated-measures analyses in individual studies when appropriate.

RevMan 5.4 was used to conduct the meta-analysis. Statistical significance was set at *p* < 0.05.

### 2.5. Quality of Assessment

Two independent reviewers assessed the methodological quality. Each included study was independently evaluated by these reviewers using the Cochrane Risk of Bias (RoB) 1.0 tool [24]. The RoB 1.0 tool was used because several included trials were reported before the introduction of RoB 2. This approach ensured a consistent assessment across all included studies using information available in the original trial reports. The RoB assessment was conducted separately for each outcome domain of interest within each study. Any disagreements were discussed and resolved within the review team. This RoB assessment, reported in the Results section, covered seven domains: (1) random sequence generation, (2) allocation concealment, (3) blinding of participants and personnel, (4) blinding of outcome assessment, (5) completeness of outcome data, (6) selective reporting, and (7) other potential sources of bias. Each domain was rated as low, unclear, or high RoB.

Publication bias was not formally assessed using funnel plots or statistical tests (e.g., Egger’s or Begg’s tests), as the number of included studies was below the minimum generally recommended for a reliable estimation.

## 3. Results

**Figure 1:**
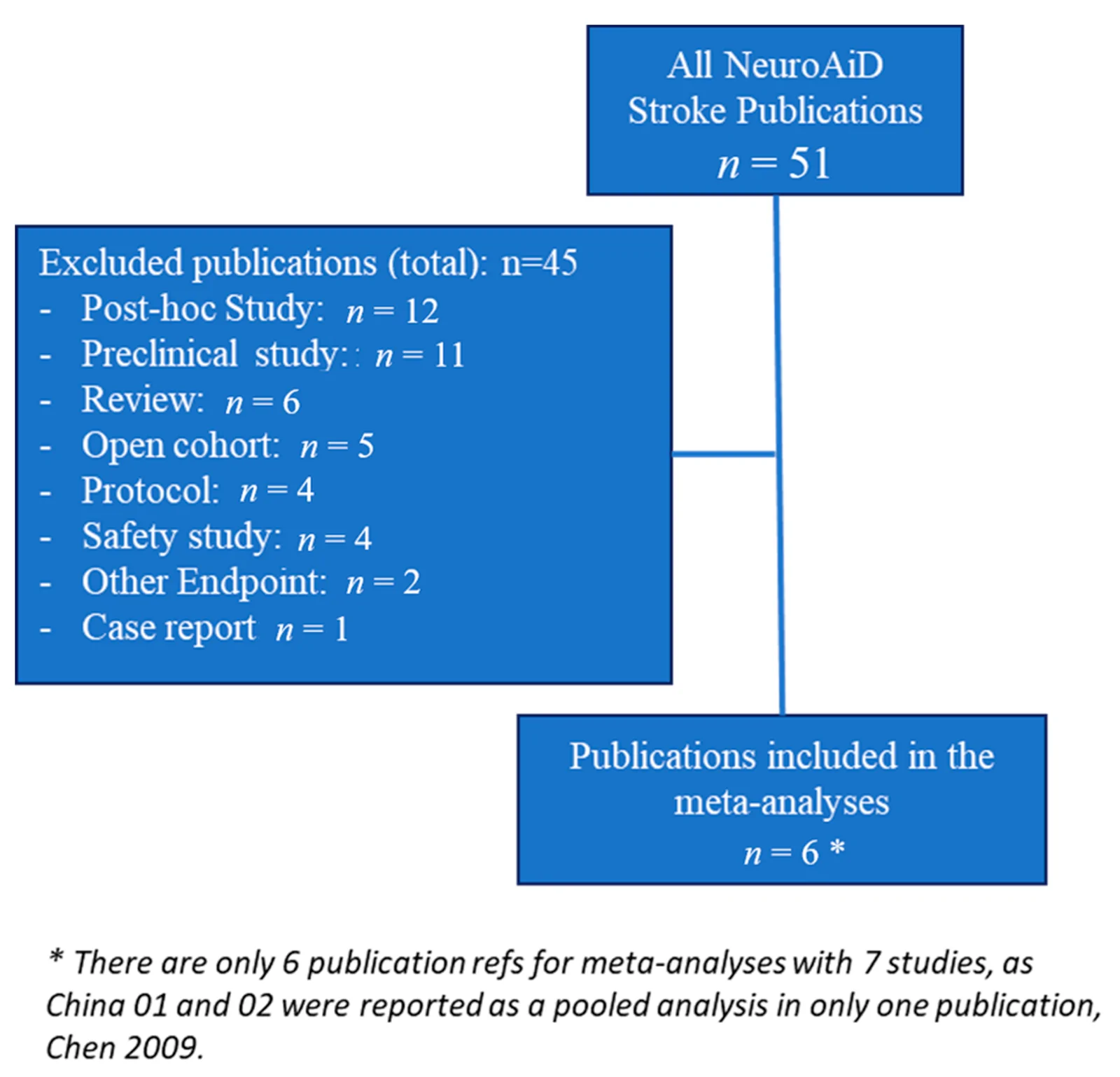
Flow Chart of NeuroAiD in ischaemic stroke clinical studies for meta-analyses on functional and motor recovery.

The search identified 90 studies, of which 51 focused on stroke. After a full-text review performed according to PRISMA criteria (Supplementary Table S1), six publications involving seven studies were included. The PRISMA flow diagram detailing the screening process is shown in Figure 1. (registered with Centre for Open Science, https://osf.io/r87gq, accessed on 1 July 2026). Table 1 displays the characteristics of the seven included studies that examined functional or motor recovery after stroke. The included studies were published between 2009 and 2015 and conducted in China, Hong Kong, Iran, Malaysia, the Philippines, Singapore, Sri Lanka, and Thailand. Sample sizes ranged from 40 to 1099, with 1535 participants assessed for functional recovery and 1774 for motor recovery at the end of the study.

### 3.1. Risk of Bias and Quality of Assessment

**Figure 2:**
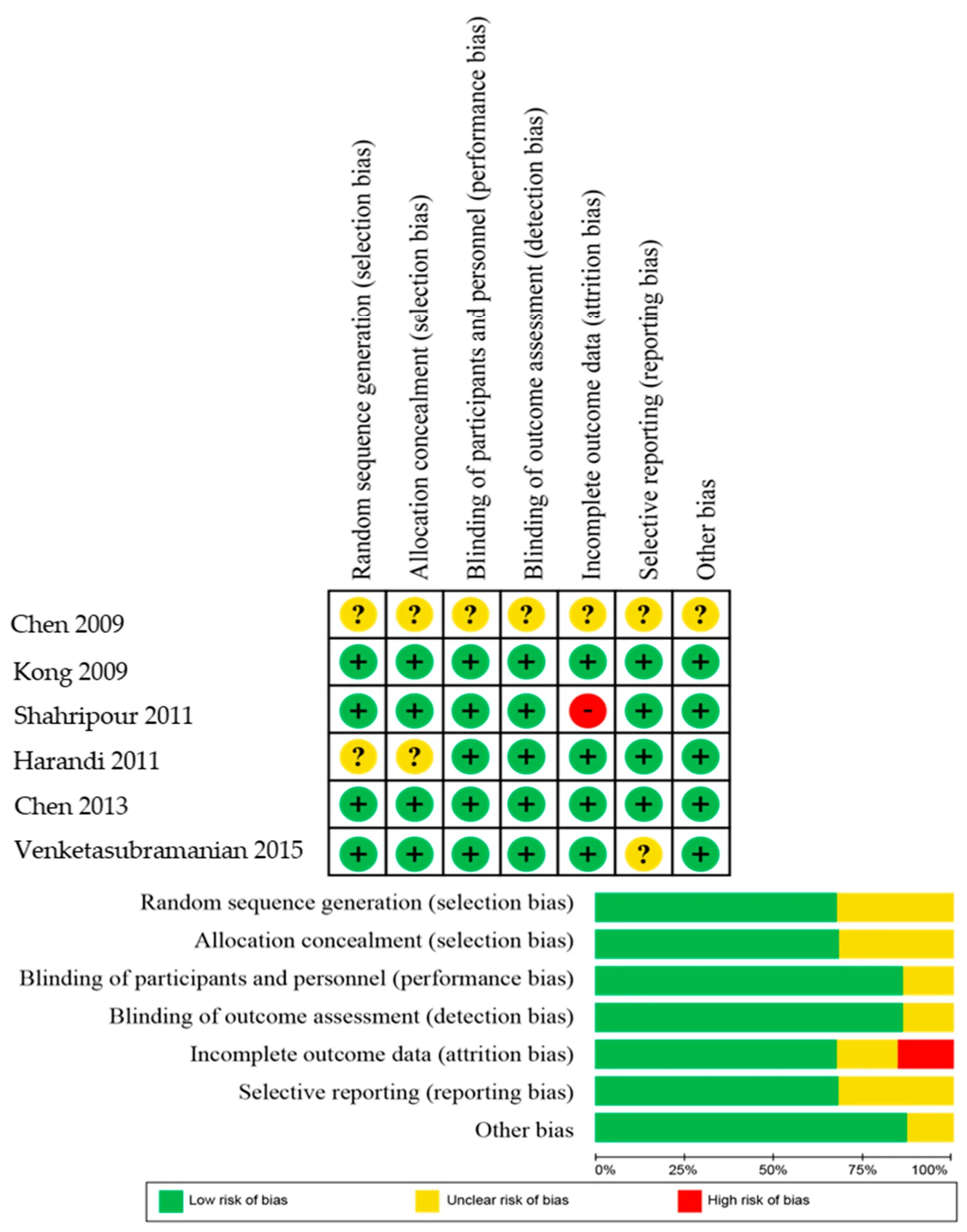
Risk of bias (RoB): judgements of the review authors for each RoB item for each included study, presented as percentages across all included studies [7,16,18,19,20,21].

As shown in Figure 2, the assessment of the seven domains of the Cochrane RoB 1.0 tool, as reported by Siddiqui et al. [15], indicated an unclear overall risk for the Chen 2009 (China 01 and 02) studies [16], with mostly low risk for the five other studies (Kong [20], Shahripour [18], Harandi [20]), and for CHIMES/CHIMES-E [7,19]. For five studies in four publications [7,16,18,19], the RoB data are sourced from Siddiqui et al. 2013 [15]. Details of the risk of bias analysis for the six selected publications [14,16,17,18,19,20] (seven studies) are given in Table 1.

### 3.2. Meta-Analyses of the Effects of Neuroaid on Long-Term Functional Recovery After Stroke 

**Figure 3:**
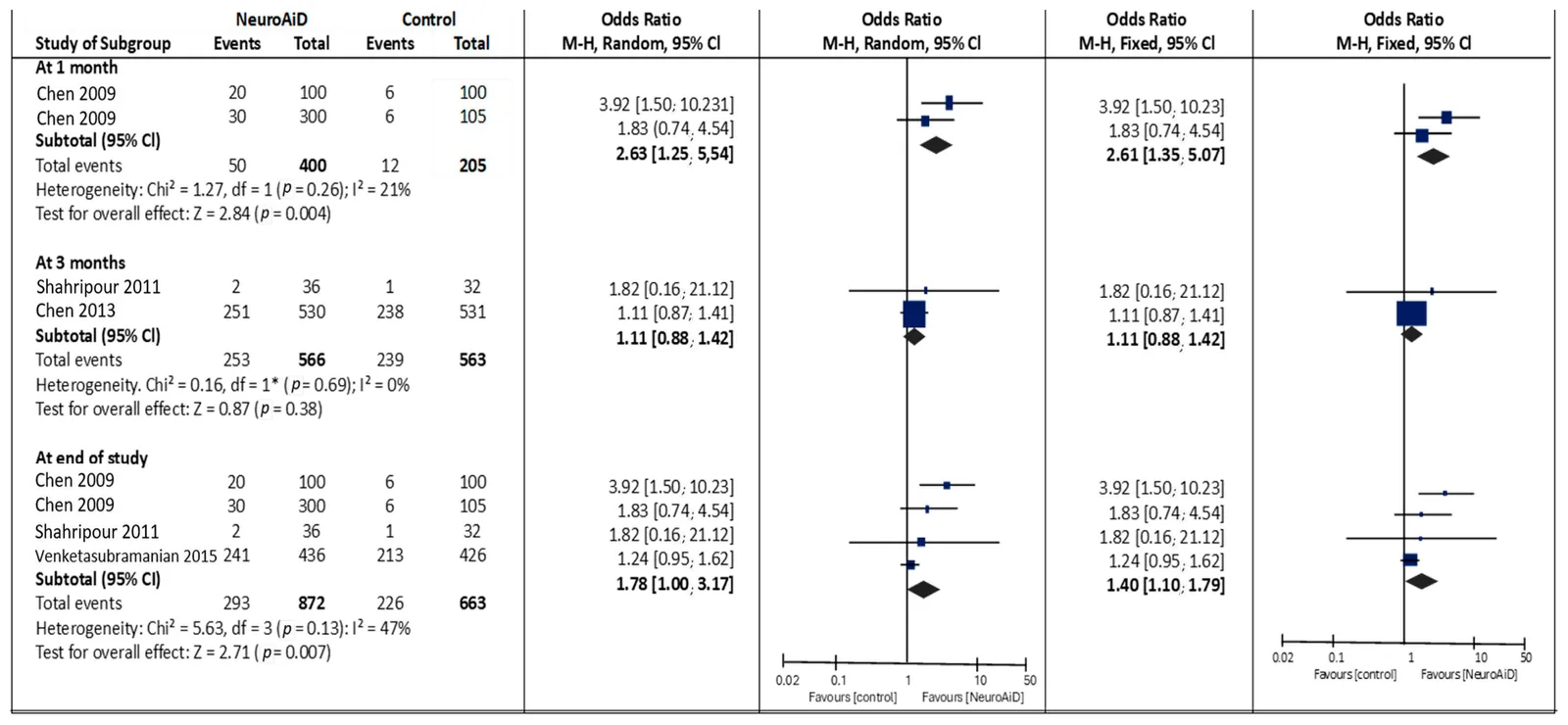
Effects of MLC601/MLC901 on long-term functional recovery after stroke—meta-analyses. M–H, Mantel–Haenszel method [7,16,18,19].

As shown in Figure 3, four studies reported functional recovery as an outcome [7,16,18,19], with a low overall risk of bias but moderate clinical heterogeneity. Functional outcomes were assessed using the DTER (item 8) in one publication of two studies [16], the BI in one study [18], and the mRS in two studies [7,19]. Both fixed-effect and random-effect models were used for the analysis; however, given the small number of included studies and the low heterogeneity observed (I^2^ values mostly at or near 0%), the results were summarised using the fixed-effect model [23]. Across follow-up time points—1 month, 3 months, 6 months, 12 months, 18 months, and 24 months—the pooled odds ratios (ORs; 95% CI) generally favoured Neuroaid over control, with statistically significant benefits observed at 1 month (OR 2.61; 1.35–5.07), 6 months (OR 1.38; 1.06–1.79), and 12 months (OR 1.33; 1.02–1.74). Although results at 3 months (OR 1.11; 0.88–1.42), 18 months (OR 1.29; 0.99–1.69) and 24 months (OR 1.24; 0.95–1.62) trended in favour of Neuroaid, no significant differences were observed.

Furthermore, pooling data from all studies at the end-of-study time point showed that Neuroaid treatment was associated with a significant improvement in functional recovery compared with control under both random- and fixed-effects models (OR 1.78; 1.00–3.17 and 1.40; 1.10–1.79, respectively). These results support the potential long-term benefits of Neuroaid in enhancing functional recovery after stroke, with effects lasting up to 24 months after treatment initiation and being most noticeable during the earlier follow-up periods.

### 3.3. Review and Meta-Analyses of the Effects of MLC601/MLC901 on Functional Recovery After Moderately Severe Stroke (b-NIHSS Score 8–14)

The CHIMES trial enrolled 1099 patients, but for this analysis, individuals with a baseline NIHSS (b-NIHSS) score of 6–7 were excluded [25]. This exclusion was based on the observation that patients with mild stroke and minimal neurological deficits tend to recover spontaneously. In the CHIMES trial, by month 3, 68% of patients in this subgroup achieved a favourable functional outcome (mRS 0–1), compared to only 32% among patients with b-NIHSS scores of 8–14 [25].

**Figure 4:**
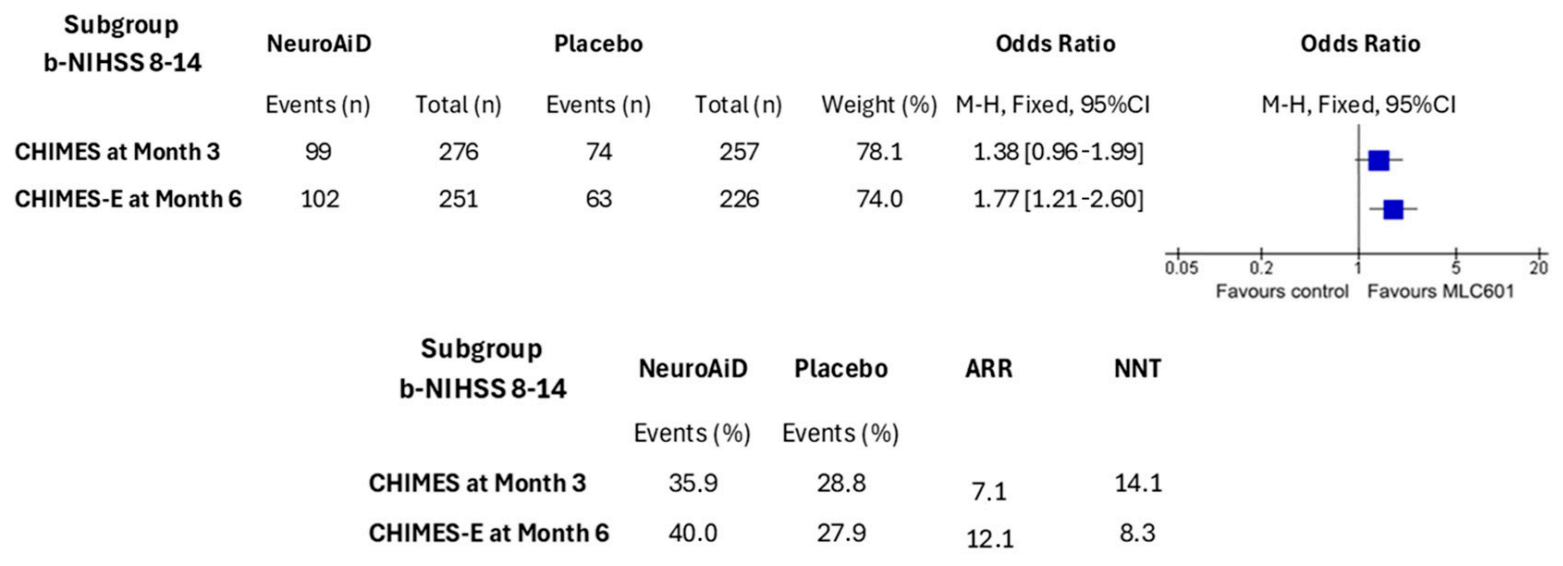
Subgroup analysis of functional recovery (mRS 0–1) in patients with a baseline NHSS (b-NIHSS) score of 8–14.

To better assess the treatment effect in a population less likely to recover without intervention, a CHIMES analysis was updated to focus only on patients with b-NIHSS 8–14. This updated subgroup analysis included CHIMES results at month 3 and CHIMES-E results at month 6, with odds ratios of 1.38 and 1.77, respectively (see Figure 4). These findings suggest that for patients with moderate baseline stroke severity (NIHSS 8–14), Neuroaid is linked with a significant and sustained improvement in functional recovery compared with standard treatment alone, with benefits evident as early as month 3 and the greatest improvement at month 6. At months 3 and 6, the numbers needed to treat (NNT) with Neuroaid were 14.1 and 8, respectively. The calculation of NNT, using success rates and absolute risk reduction (ARR), is shown in Figure 4.

### 3.4. Review and Meta-Analyses of the Effects of Neuroaid on Motor Recovery After Stroke

**Figure 5:**
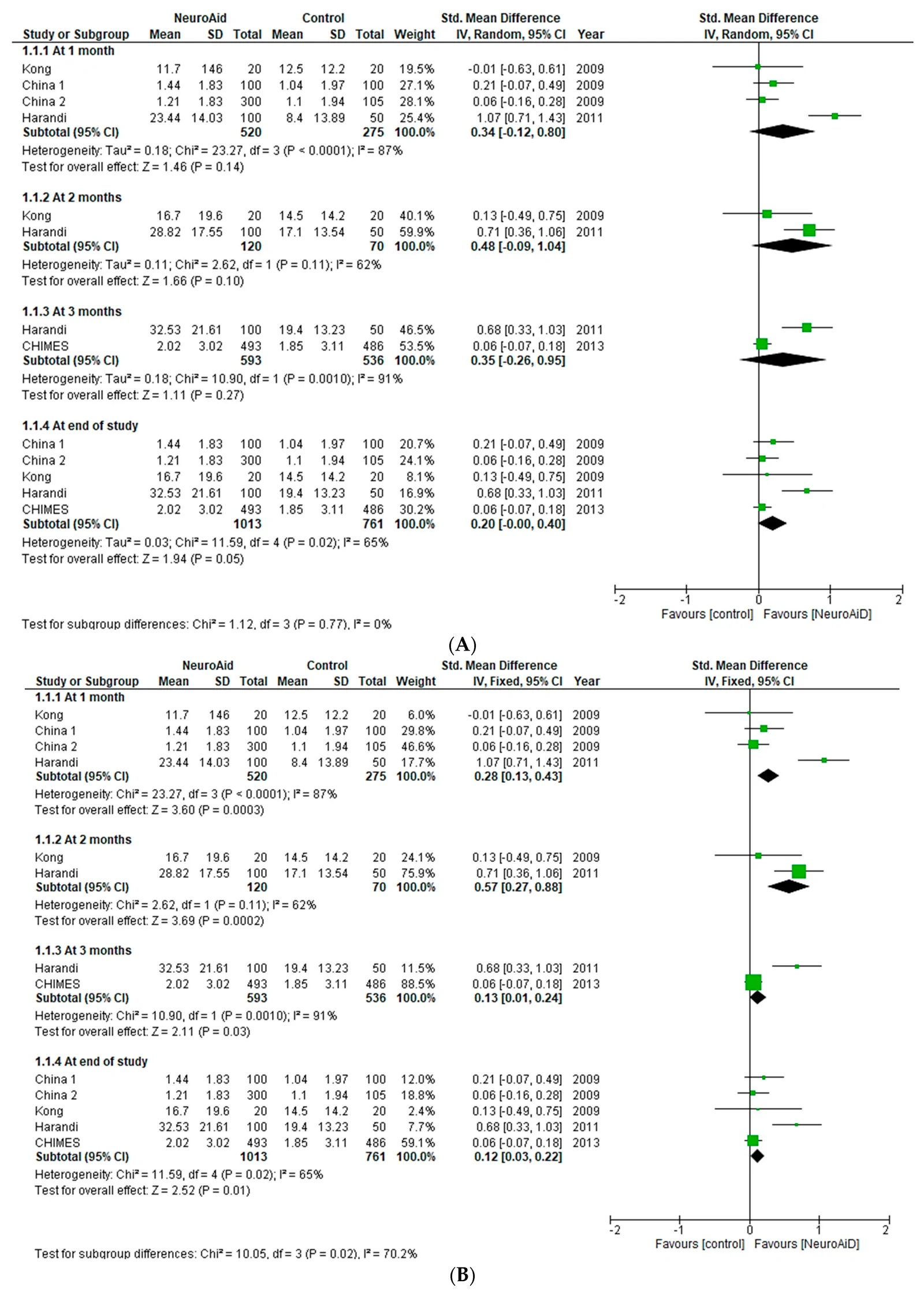
Effects of Neuroaid on motor recovery after stroke—meta-analyses with (**A**) random-effects model, (**B**) fixed-effects model [7,6,20,21].

As shown in Figure 5, this review of motor recovery trials included five controlled studies [7,16,19,20,21] that had an overall low risk of bias but moderate heterogeneity. Motor recovery outcomes were assessed using validated scales: the DTER motor scores in two studies, the FMA motor score in two studies, and the NIHSS motor score in one study. Since outcomes were reported as continuous data on different scales, standardised mean differences were used for meta-analysis.

Pooled analyses were conducted at several follow-up time points, and both fixed-effects (FE) and random-effects (RE) models were used for exploration [22] (Figure 5):

• 1 month (four studies, *n* = 795): SMD = 0.28 (95% CI 0.13–0.43, FE) and 0.34 (95% CI −0.12–0.80, RE), indicating a small-to-moderate, statistically significant benefit in the FE model.
• 2 months (two studies, *n* = 190): SMD = 0.57 (95% CI 0.27–0.88, FE) and 0.48 (95% CI −0.09–1.04, RE), indicating a moderate effect size that was significant in the FE model.
• 3 months (two studies, *n* = 1129): SMD = 0.13 (95% CI 0.01–0.24, FE) and 0.35 (95% CI −0.26–0.95, RE), reflecting a small but significant effect in the FE model.
• End of study (five studies, *n* = 1774): SMD = 0.12 (95% CI 0.03–0.22, FE) and 0.20 (95% CI 0.00–0.40, RE), showing a consistent benefit across both models, with statistical significance in the FE model.

The pooled effect favoured Neuroaid over control for up to 24 months, and at the end of the study. These findings support using Neuroaid as an adjunct to standard care to improve motor recovery in patients with primarily subacute, stable stroke. The Neuroaid effect is most pronounced during the first two months of treatment and persists at the end of follow-up, supporting its potential role in long-term rehabilitation strategies.

## 4. Discussion

This comprehensive review and series of meta-analyses, drawing exclusively on RCTs that used validated, standardised outcome measures, provides consistent evidence supporting MLC601/MLC901 (NeuroAiD) as an effective supplement to standard care for improving both functional and motor recovery after stroke. These findings have significant clinical implications for post-stroke rehabilitation strategies. The consistent benefits across both functional and motor outcomes support that Neuroaid can effectively supplement standard therapy, potentially improving independence and quality of life for stroke survivors. In particular, the robust effects in patients with moderate stroke severity highlight a subgroup that may benefit the most from the intervention. The observed early and sustained improvements also suggest that starting MLC601 early in the rehabilitation process may maximise recovery potential, particularly for motor outcomes, where neuroplastic changes are most responsive in the first weeks and months after stroke [26,27].

Across multiple analyses, MLC601 demonstrated significant benefits in functional recovery at several time points. Its beneficial effects on long-term functional recovery are supported by significant ORs with MLC601 at 1, 6, and 12 months, with pooled end-of-study results showing a 40% higher likelihood of a favourable outcome than in the control group. Although the pooled estimates for functional recovery at 3, 18, and 24 months favoured MLC601, the corresponding confidence intervals crossed unity, supporting a favourable trend, albeit not reaching statistical significance. At the longer follow-up time points, this may reflect increasing uncertainty due to attrition, reduced sample sizes, and the limited number of studies contributing long-term data. At 3 months, the absence of a statistically significant overall effect may be partly explained by the inclusion of patients with milder strokes, who have a high rate of spontaneous recovery and may therefore dilute treatment effects (indeed, we have previously reported that patients with an initial NIHSS score of 6 to 7 had limited symptoms and tended to recover spontaneously: at 3 months, 68% of these patients had recovered in this subgroup, while only 32% in the rest of the CHIMES population (initial NIHSS of 8 to 14) had recovered [25]). In CHIMES, about 50% of the patients had a stroke of mild severity (NIHSS < 8). Similarly, for motor recovery, significant findings observed under the fixed-effects model at 1, 2, and 3 months were not consistently maintained under the random-effects model, reflecting the limited number of studies and heterogeneity in patient characteristics, rehabilitation approaches, and outcome measures. These findings suggest that while the overall direction of effect consistently favoured MLC601, the precise magnitude of this benefit may vary across contexts and should be interpreted with caution.

In the moderately severe stroke subgroup (b-NIHSS 8–14), consistent and statistically significant improvements were seen at months 3 (OR 1.64) and 6 (OR 1.98), indicating a strong and sustained benefit in this population. For motor recovery, standardised mean differences indicated small-to-moderate, statistically significant improvements in the fixed-effects model at all measured time points (1, 2, 3 months, and end of study). The most significant effects emerge in the first two months, indicating a critical early treatment window [28]. There is some loss of statistical significance under the random-effects model at 1, 2 and 3 months. This may be due to the relatively small number of studies and some heterogeneity between the various trials. Despite this heterogeneity, the statistical consequence was generally low for functional recovery (I^2^ mostly ≈0%), resulting in consistent estimates under both fixed- and random-effects models. For motor recovery, heterogeneity was moderate to substantial (I^2^ 62–91%), reflecting differences in study populations, rehabilitation protocols, assessment timing, and outcome measures. Although this increased uncertainty and widened confidence intervals under the random-effects model, the direction of the treatment effect consistently favoured NeuroAiD across all analyses. Compared with previously published systematic reviews and meta-analyses [7], the present study offers a more comprehensive and clinically relevant evaluation of MLC601/MLC901 by incorporating additional randomised controlled trials with a significantly larger patient cohort, and longer-term follow-up data (up to 24 months) [19]. Specific analyses of motor recovery were conducted using validated scales, including the Fugl–Meyer Assessment and the NIHSS motor score [6]. Importantly, this analysis also includes patients with moderately severe stroke (baseline NIHSS score of 8–14), a subgroup with lower spontaneous recovery potential and greater unmet rehabilitation needs [25]. Taken together, these findings strengthen the evidence that MLC601/MLC901 provides lasting benefits in both functional independence and motor recovery when used alongside standard post-stroke care.

Clinical decisions for stroke rehabilitation in recovery centres are often based on the healthcare team’s clinical judgement, taking into account factors such as stroke severity, age, and sometimes the patient’s social support network [29]. While this personalised approach helps tailor care for each patient, it can also lead to variability in care planning across clinicians, institutions, and clinical studies. Without standardised assessment tools, prognosis and rehabilitation strategies can vary widely, potentially leading to inconsistent results and unequal access to the best recovery options. Standardised, validated assessment tools can enhance consistency and fairness in decision-making [30]. In stroke rehabilitation, two key domains are commonly assessed: functional independence and motor function. Functional independence is generally measured using the mRS, while motor recovery is evaluated using validated tools such as the FMA or other structured motor scoring systems.

For functional recovery, the mRS provides a global measure of disability, ranging from no symptoms (score 0) to severe disability (score 5) or death (score 6). It is commonly used to assess independence in daily activities, classify recovery trajectories, guide discharge planning, and evaluate treatment effectiveness. However, rehabilitation planning often benefits from tools that predict specific functional abilities rather than broad positive or negative outcomes [30].

These results confirm the findings from two clinical studies evaluating the effects of Neuroaid on functional recovery. In patients who continued rehabilitation through month 3 (M3), adding MLC601 to rehabilitation increased the likelihood of improved functional recovery and independence at 3 months and beyond compared with placebo in well-balanced groups [31]. These findings support a beneficial and sustained effect on neurorepair processes following acute ischaemic stroke. The beneficial effect of treatment is found across various outcome measures, including mRS dichotomy and BI. Across these measures, the treatment effect consistently leads to a greater proportion of patients achieving recovery or functional independence than rehabilitation alone. Importantly, this clinical benefit appears to persist for up to two years, as reflected by a stable NNT over time. The other study, EPICA, was conducted in Spain to examine, using multivariate analyses, whether the effect of MLC901 varied by stroke severity [32]. At month 3, both groups (MLC901 and control) showed improved scores, with no statistically significant differences. However, a higher proportion of patients receiving MLC901 achieved mRS scores above the median than controls (75.4% vs 66.7%; *p* = 0.25). A slight trend favouring MLC901 was also observed in NIHSS outcomes (51% vs. 48%). To further explore stroke severity as a potential confounder, outcomes were stratified by baseline NIHSS score, classifying strokes as moderate (NIHSS ≤ 14; *n* = 96) or more severe (NIHSS > 14; *n* = 35). Among patients with more severe stroke, a greater proportion treated with MLC901 achieved above-median improvements in mRS at month 1 (*p* = 0.032) and month 3 (*p* = 0.058) compared with controls.

Post-stroke motor deficits can affect the upper limbs, lower limbs, or both, depending on the lesion location [33]. Several validated scales are commonly used in clinical settings to measure motor impairment (Table 2).

One of the most commonly used is the FMA score, which evaluates motor recovery, sensation, balance, joint motion, and pain [34]. Although comprehensive, the full FMA is time-consuming (about 30–45 min) and may be less sensitive to subtle improvements in higher-functioning patients. A commonly used subscale, the FMA motor score, focuses specifically on motor function and demonstrates strong inter-assessor reliability [35]. Other measures include the Functional Independence Measure (FIM) Motor Subscale, which assesses motor ability but is less specific to neurological recovery and can be affected by compensatory strategies [36]. The NIHSS is widely used to evaluate stroke severity and guide urgent treatment decisions. Its baseline Limb Motor Score (b-NIHSS-LMS), which combines arm and leg scores, has been linked to functional recovery at 3 months post-stroke [6]. Together, the mRS and standardised motor assessments provide a more comprehensive understanding of recovery potential, supporting evidence-based rehabilitation planning and improving comparability of outcomes across clinical settings [37].

These results confirm the findings from two individual studies of MLC601 on motor recovery, assessed with the FMA [20,21], which provide additional evidence that Neuroaid improves post-stroke motor recovery. In the double-blind, placebo-controlled pilot study by Kong et al. [20], 40 patients with ischaemic stroke within 1 month of onset were randomised to receive either MLC601 or a placebo (four capsules, three times daily for 4 weeks). Outcomes included FMA, NIHSS, and FIM scores at baseline, 4 weeks, and 8 weeks. While most endpoints showed no statistically significant difference versus placebo, FMA scores showed a positive trend over time with MLC601, particularly in patients with posterior circulation strokes and more severe deficits. The Harandi et al. trial provided stronger evidence. In this randomised, placebo-controlled study, FMA scores were assessed at baseline and at 4, 8, and 12 weeks in 150 patients [21]. At baseline, scores were comparable between study groups (MLC601: 53.69 ± 23.01 vs. placebo: 54.96 ± 24.27; *p* = 0.755). However, by 4 weeks, patients in the NeuroAiD group had significantly higher FMA scores (77.13 ± 19.22 vs. 63.50 ± 24.21; *p* < 0.001), with differences persisting and widening at 8 weeks (82.51 ± 14.27 vs. 72.06 ± 21.41; *p* = 0.001) and 12 weeks (86.22 ± 12.34 vs. 74.36 ± 18.10; *p* < 0.001) compared to the placebo group. Repeated-measures analysis confirmed a statistically significant difference in overall FMA scores between MLC601 and placebo groups (*p* = 0.003), with a strong time effect (*p* < 0.0001). The most noticeable improvements appeared after the first month and continued throughout the 12-week period. Overall, both studies show that MLC601 speeds up and enhances motor recovery after stroke, with measurable, statistically significant improvements in FMA scores as early as 4 weeks and sustained through 12 weeks. The greatest functional gains occur during the first month, supporting early treatment alongside rehabilitation.

To provide a balanced view of benefits and harms, here is a summary of safety data from the included studies. The available clinical evidence indicates that MLC601/MLC901 has a favourable safety profile in patients with ischaemic stroke, both in the acute and recovery phases. In the CHIMES trial involving 1099 patients with acute ischemic stroke, MLC601 was well tolerated and was associated with fewer serious adverse events (SAEs) than placebo (60 vs. 75 patients), fewer recurrent SAEs, and significantly lower rates of prolonged hospitalisation, with no new safety concerns identified [38]. A systematic review of six clinical studies further concluded that MLC601 is safe as an adjunct to standard stroke treatment, reporting no deaths and only four serious adverse events among treated patients, without any consistent safety signals. Similarly, in the double-blind, placebo-controlled trial involving 150 Iranian stroke patients, treatment was well tolerated, with only mild and transient nausea and vomiting reported in seven patients, no clinically significant abnormalities in haematological, hepatic, or renal parameters, and no treatment discontinuations due to adverse effects. Earlier randomised studies and pooled analyses also demonstrated excellent tolerability, with no major concerns related to haemostasis, haematology, biochemistry, or bleeding risk. Overall, the evidence supports the safety and tolerability of MLC601 when used alongside standard stroke care.

It is important to note that the decision to conduct a subgroup meta-analysis on patients with moderate baseline stroke severity (b-NIHSS 8–14) addressed a clinically relevant question. By excluding those with mild strokes (b-NIHSS 6–7), who have a high probability of spontaneous recovery, the analysis avoided dilution of treatment effects and more accurately reflected the clinically meaningful impact of MLC601 in patients with greater rehabilitation needs.

Some limitations should be recognised. Although we believe all relevant MLC601/MLC901 stroke trials were identified, the absence of formal searches of additional databases such as Embase and Cochrane CENTRAL remains a limitation. Although most studies demonstrated a low risk of bias according to the Cochrane assessment, some publication bias cannot be excluded. Because the number of included studies was small, conventional methods such as funnel plots, Egger’s test, and Begg’s test would have limited reliability. Consequently, the possibility that unpublished negative studies exist cannot be formally excluded. In addition, some of the included studies received some level of support from the manufacturer of NeuroAiD. These factors may have influenced the evidence base and should be considered when interpreting the findings. This review was not prospectively registered in PROSPERO. This could potentially raise questions regarding the use of predefined review methods. The certainty of evidence was not formally assessed using the GRADE framework. Consequently, although the included studies were only randomised controlled trials with generally low risk of bias, the overall strength of evidence should be interpreted with caution, particularly given the limited number of studies available for several analyses. Because all included studies were conducted in Asia or the Middle East, caution is warranted when extrapolating the findings to populations with different ethnic, genetic, healthcare-system, or rehabilitation characteristics. Heterogeneity in standard stroke management, the nature and intensity of rehabilitation programmes, healthcare resources, and clinical practices across countries and study centres may have influenced recovery outcomes and affected the estimated magnitude of the treatment effect.

Another limitation is the heterogeneity of outcome measures across the included studies. Functional recovery was assessed using different validated scales (mRS, Barthel Index, and DTER), while motor recovery was evaluated using the Fugl–Meyer Assessment, NIHSS motor score, or DTER motor items. Although these instruments measure related but not identical constructs, all are validated and widely used in stroke rehabilitation. To accommodate this methodological heterogeneity, standardised meta-analytic approaches were applied, including standardised mean differences for continuous outcomes. Nevertheless, differences in outcome measures may reduce the direct clinical interpretability of the pooled effect estimates and should be considered when interpreting the findings.

Furthermore, although the functional benefits remained consistent, the clinical significance of small SMD values for motor recovery merits further investigation in pragmatic trials. So, it must be evaluated with the understanding that even modest improvements in motor function may be clinically meaningful after stroke, particularly when observed across multiple domains, and when considering that these relatively modest motor effects help facilitate the conduct and maximise the benefit of active rehabilitation. Because treatment effects were evaluated at multiple follow-up time points without formal adjustment for multiplicity, the statistical significance of a *p*-value <0.05 should be interpreted with caution. However, the overall conclusions of this review are supported by the consistency of the direction of treatment effects across multiple studies, functional and motor outcome measures, and different follow-up periods, rather than by isolated statistically significant comparisons. The ability to investigate treatment effect across subgroups was limited by the availability of published data. Apart from the predefined analysis of patients with baseline NIHSS scores of 8–14, additional subgroup meta-analyses could not be performed. Future individual-data meta-analyses would allow for a more robust assessment of potential effect modifiers.

The study has several strengths that support the reliability and relevance of its findings. First, it includes only RCTs, which provide the highest level of clinical evidence. Limiting the analysis to RCTs comparing Neuroaid with placebo or active comparator reduces confounding and strengthens the internal validity of the results. A systematic search strategy and clearly defined inclusion criteria also ensured a transparent and comprehensive synthesis of evidence. Another strength is the large pooled sample size. The meta-analysis included six publications reporting seven studies, with over 1500 participants for functional outcomes and over 1700 for motor outcomes. This enhances statistical power and the accuracy of treatment effect estimates. The studies were conducted across several Asian countries, increasing the generalisability of the findings to diverse clinical settings. The analysis also used widely validated outcome measures, including the mRS, BI, FMA motor score, and NIHSS motor scores. Using multiple standardised scales enabled a comprehensive assessment of both functional independence and motor recovery. Finally, the study used robust statistical methods, including both fixed- and random-effects models, and evaluated study quality with the Cochrane Risk of Bias tool. Using random-effects models as the primary analytical method is generally considered the most suitable framework for meta-analyses because it accounts for potential variability across studies. Given the low statistical heterogeneity observed across most datasets (I^2^ close to 0% in the functional recovery analysis), results from fixed-effect models were also reported to provide complementary estimates with greater precision [39]. As expected, random-effects analyses yielded wider confidence intervals in some instances, reducing statistical significance at certain time points, particularly when fewer studies were included [40]. These parallel analyses support the robustness of the findings.

## 5. Conclusions

The available evidence suggests that MLC601/MLC901 is associated with functional and motor recovery after ischemic stroke when used in addition to standard care. Although all randomised studies of MLC601/MLC901 in stroke have been included in this meta-analysis, it must be recognised that the number of trials remains modest (seven studies), and that the certainty of the evidence was judged to be moderate at best in the absence of a formal GRADE assessment. Further large, independent, multinational randomised trials would strengthen the evidence base and refine estimates of treatment effect. Benefits are most pronounced in patients with moderate stroke severity and appear as early as the first month, with sustained effects up to two years. Fugl–Meyer Assessment data from individual trials further confirm its positive impact on motor recovery, supporting early initiation alongside rehabilitation to maximise neuroplastic potential. These findings provide strong evidence that MLC601/MLC901 is a valuable addition to post-stroke treatment and rehabilitation strategies, enhancing independence and quality of life.

## Figures and Tables

**Table 1:** Baseline characteristics of the studies included in the meta-analyses.

First Author	Year	Country	Sample Size	Stroke Type	Stroke Severity	Intervention (Dose, Duration)	Comparator	Outcomes	Follow-Up Duration	Risk of Bias Assessment
Chen [16]	2009	China	200 + 405	AIS	DTER score ≥ 10 from mild to severe	Danqi Piantang Jiaonang (DPJ/MLC601). four capsules after each meal, 3 times per day for 4 weeks.	Buchang Naoxintong Jiaonang (BNJ)	Functional outcome, measured by the Comprehensive Function Score component of the Diagnostic Therapeutic Effects of Apoplexy (DTER) scale	1 month	Unclear
Kong [20]	2009	Singapore	40	Less than 1-month ischaemic stroke	Baseline NIHSS: 6.25	MLC601 4 capsules 3 times daily 1-month treatment	Placebo	Improvement of motor impairment of the affected upper and lower limbs on the Fugl- Meyer Assessment (FMA) at 4 weeks	1 month	Low
Shahripour [18]	2011	Iran	80	1-week ischaemic stroke	Baseline Barthel Index (BI) mean: 35.6	MLC601 4 capsules 3 times daily 3-month treatment	Placebo	BI score of 65 as a cut-off for the level of desired dependency	3 months	Low = 6 & High = 1
Harandi [21]	2011	Iran	150	Less than 1-month ischaemic stroke	Based on baseline Fugl- Meyer Assessment (FMA) score and days since stroke	MLC601 4 capsules 3 times daily 3-month treatment	Placebo	Improvement of motor impairment of the affected upper and lower limbs on the FMA at baseline, 4, 8, and 12 weeks after stroke	12 weeks	Low = 5 & Uncertain = 2
Chen [7]	2013	CHIMES Study: Philippines, Singapore, Thailand, Sri Lanka, Hong Kong, Malaysia	1099	Patients with the National Institutes of Health Stroke Scale (NIHSS) score 6 to 14, within 72 h of onset.	Baseline NIHSS (6–14): 8.7	MLC601 4 capsules 3 times daily 3-month treatment	Placebo	Primary outcome: shift in the modified Rankin Scale (mRS). Secondary outcomes: mRS dichotomy	3 months	Low = 7
Venketasubramanian [19]	2015	CHIMES-E Study: Singapore, Philippines, Thailand, Hong Kong, Malaysia	880	All Subjects were randomised to either MCL601 or a placebo in CHIMES were eligible for inclusion in CHIMES-E:	Baseline NIHSS: 8.6	MLC601 was stopped after the initial 3-month course of the CHIMES study	Placebo was stopped after the initial 3-month course of the CHIMES study	Effects of the initial 3-month treatment with MLC601 on long-term outcome for up to 2 years.	24 months	Low = 6 & Uncertain = 1

**Table 2:** Characteristics of scales to assess motor function and impairment.

Scale	Main Focus	Score Range	Time to Administer	Best For
FMA	Comprehensive motor, sensory, balance, range of motion, and pain	0–226 total (Motor: 0–100)	30–45 min	Gold standard impairment assessment
FMA Motor	Motor impairment only	0–100	20–30 min	Detailed motor recovery monitoring
FIM Motor	Functional independence in daily tasks	13–91	20–30 min	Rehabilitation planning and monitoring functional outcomes
NIHSS Motor	Acute stroke motor severity	Arm/leg items 0–4 each	5–10 min	Acute stroke severity grading

FMA: Fugle-Meyer Assessment; FIM: Functional Independence Measure; NIHSS: National Institutes of Health Stroke Scale.

## Data Availability

All data generated or analysed during this review are included in the article, along with references to data from cited published studies. Further inquiries can be directed toward the corresponding author.

## Supplementary Materials

The following supporting information can be downloaded at: https://www.mdpi.com/article/10.3390/neurolint18080141/s1, Table S1: PRISMA 2020 Checklist. Reference [41] are cited in the supplementary materials.
