# Supplementary material for: Review and Meta-Analyses of the Effects of MLC601/MLC901 (NeuroAiD) on Post-Stroke Functional and Motor Recovery
Source: Neurol Int. 2026 Jul 23;18(8):141. doi: 10.3390/neurolint18080141 (PMC13516714; doi:10.3390/neurolint18080141)
Supplement: Supplementary file 1 [file neurolint-18-00141-s001.zip › neurolint-4383254-Table S1-PRISMA_2020_checklist_completed.pdf]

PRISMA 2020 Checklist

Section and Topic	Item #	Checklist item	Location where item is reported
**TITLE**			
Title	1	Identify the report as a systematic review.	Title page: manuscript title identifies the report as a review and meta-analyses.
**ABSTRACT**			
Abstract	2	See the PRISMA 2020 for Abstracts checklist.	Abstract: Background, Methods, Results, Conclusions.
**INTRODUCTION**			
Rationale	3	Describe the rationale for the review in the context of existing knowledge.	Introduction: rationale for post-stroke recovery need and existing NeuroAiD evidence.
Objectives	4	Provide an explicit statement of the objective(s) or question(s) the review addresses.	Methods, section 1: Objectives.
**METHODS**			
Eligibility criteria	5	Specify the inclusion and exclusion criteria for the review and how studies were grouped for the syntheses.	Methods, section 2: Data Sources and Selection Criteria.
Information sources	6	Specify all databases, registers, websites, organisations, reference lists and other sources searched or consulted to identify studies. Specify the date when each source was last searched or consulted.	Methods, section 2: Data Sources and Selection Criteria (PubMed searched up to 31 December 2025).
Search strategy	7	Present the full search strategies for all databases, registers and websites, including any filters and limits used.	Methods, section 2: Data Sources and Selection Criteria (key PubMed search terms reported; full line-by-line strategy not reported).
Selection process	8	Specify the methods used to decide whether a study met the inclusion criteria of the review, including how many reviewers screened each record and each report retrieved, whether they worked independently, and if applicable, details of automation tools used in the process.	Methods, section 2: Data Sources and Selection Criteria; Results: Study selection; Figure 1.
Data collection process	9	Specify the methods used to collect data from reports, including how many reviewers collected data from each report, whether they worked independently, any processes for obtaining or confirming data from study investigators, and if applicable, details of automation tools used in the process.	Methods, section 3: Data extraction.
Data items	10a	List and define all outcomes for which data were sought. Specify whether all results that were compatible with each outcome domain in each study were sought (e.g. for all measures, time points, analyses), and if not, the methods used to decide which results to collect.	Methods, sections 2 and 3: Data Sources and Selection Criteria; Data extraction.
	10b	List and define all other variables for which data were sought (e.g. participant and intervention characteristics, funding sources). Describe any assumptions made about any missing or unclear information.	Methods, section 3: Data extraction (study, baseline, treatment, and outcome variables listed; assumptions for missing/unclear information not reported).
Study risk of bias assessment	11	Specify the methods used to assess risk of bias in the included studies, including details of the tool(s) used, how many reviewers assessed each study and whether they worked independently, and if applicable, details of automation tools used in the process.	Methods, section 5: Quality of assessment; Results, section 1; Figure 2; Supplementary Table 1.
Effect measures	12	Specify for each outcome the effect measure(s) (e.g. risk ratio, mean difference) used in the synthesis or presentation of results.	Methods, section 4: Statistical Analyses.

PRISMA 2020 Checklist

Section and Topic	Item #	Checklist item	Location where item is reported
Synthesis methods	13a	Describe the processes used to decide which studies were eligible for each synthesis (e.g. tabulating the study intervention characteristics and comparing against the planned groups for each synthesis (item #5)).	Methods, sections 2 and 4: Data Sources and Selection Criteria; Statistical Analyses.
	13b	Describe any methods required to prepare the data for presentation or synthesis, such as handling of missing summary statistics, or data conversions.	Methods, sections 3 and 4: Data extraction; Statistical Analyses.
	13c	Describe any methods used to tabulate or visually display results of individual studies and syntheses.	Results: Figure 1, Table 1, Figure 2, Supplementary Table 1, Figures 3-5, Table 2.
	13d	Describe any methods used to synthesize results and provide a rationale for the choice(s). If meta-analysis was performed, describe the model(s), method(s) to identify the presence and extent of statistical heterogeneity, and software package(s) used.	Methods, section 4: Statistical Analyses.
	13e	Describe any methods used to explore possible causes of heterogeneity among study results (e.g. subgroup analysis, meta-regression).	Methods, section 4: Statistical Analyses; Results, section 3: baseline NIHSS 8-14 subgroup analysis; Figure 4.
	13f	Describe any sensitivity analyses conducted to assess robustness of the synthesized results.	Methods, section 4: Statistical Analyses (sensitivity analyses with random-effects models when possible).
Reporting bias assessment	14	Describe any methods used to assess risk of bias due to missing results in a synthesis (arising from reporting biases).	Not reported: no specific methods for assessing reporting bias or missing results in syntheses were described.
Certainty assessment	15	Describe any methods used to assess certainty (or confidence) in the body of evidence for an outcome.	Not reported: no formal certainty/confidence assessment method, such as GRADE, was described.
**RESULTS**			
Study selection	16a	Describe the results of the search and selection process, from the number of records identified in the search to the number of studies included in the review, ideally using a flow diagram.	Results: Study selection; Figure 1.
	16b	Cite studies that might appear to meet the inclusion criteria, but which were excluded, and explain why they were excluded.	Not reported in itemised form: excluded study categories are described in Methods, section 2, but excluded full-text studies are not individually cited with reasons.
Study characteristics	17	Cite each included study and present its characteristics.	Results: Study selection; Table 1: Baseline characteristics of included studies.
Risk of bias in studies	18	Present assessments of risk of bias for each included study.	Results, section 1: Risk of bias and Quality of assessment; Figure 2; Supplementary Table 1.
Results of	19	For all outcomes, present, for each study: (a) summary statistics for each group (where appropriate) and (b) an effect estimate and	Results, sections 2-4; Figures 3-5; Discussion

PRISMA 2020 Checklist

Section and Topic	Item #	Checklist item	Location where item is reported
individual studies		its precision (e.g. confidence/credible interval), ideally using structured tables or plots.	paragraphs summarising individual FMA trials.
Results of syntheses	20a	For each synthesis, briefly summarise the characteristics and risk of bias among contributing studies.	Results, sections 2 and 4: descriptions of contributing studies and overall risk of bias.
	20b	Present results of all statistical syntheses conducted. If meta-analysis was done, present for each the summary estimate and its precision (e.g. confidence/credible interval) and measures of statistical heterogeneity. If comparing groups, describe the direction of the effect.	Results, sections 2-4; Figures 3-5.
	20c	Present results of all investigations of possible causes of heterogeneity among study results.	Results, section 3: moderately severe stroke subgroup analysis; Figure 4.
	20d	Present results of all sensitivity analyses conducted to assess the robustness of the synthesized results.	Results, section 4 and Discussion: fixed- and random-effects model comparisons; sensitivity analyses referenced in Methods.
Reporting biases	21	Present assessments of risk of bias due to missing results (arising from reporting biases) for each synthesis assessed.	Not reported: no assessment of risk of bias due to missing results/reporting biases is presented.
Certainty of evidence	22	Present assessments of certainty (or confidence) in the body of evidence for each outcome assessed.	Not reported: no formal certainty/confidence ratings by outcome are presented.
**DISCUSSION**			
Discussion	23a	Provide a general interpretation of the results in the context of other evidence.	Discussion: interpretation of functional and motor recovery findings in context of prior evidence.
	23b	Discuss any limitations of the evidence included in the review.	Discussion: limitations paragraph on heterogeneity, dosing duration, rehabilitation protocols, and motor SMD clinical significance.
	23c	Discuss any limitations of the review processes used.	Discussion: limitations paragraph; review-process limitations only partly addressed.
	23d	Discuss implications of the results for practice, policy, and future research.	Discussion and Conclusions: implications for post-stroke rehabilitation and early initiation alongside standard care.
**OTHER INFORMATION**			
Registration and protocol	24a	Provide registration information for the review, including register name and registration number, or state that the review was not registered.	Not reported: registration information or statement that the review was not registered is absent.

PRISMA 2020 Checklist

Section and Topic	Item #	Checklist item	Location where item is reported
	24b	Indicate where the review protocol can be accessed, or state that a protocol was not prepared.	Not reported: protocol availability or statement that no protocol was prepared is absent.
	24c	Describe and explain any amendments to information provided at registration or in the protocol.	Not reported / not applicable: no registration or protocol amendments described.
Support	25	Describe sources of financial or non-financial support for the review, and the role of the funders or sponsors in the review.	Acknowledgements/Funding: “This research received no external funding.”
Competing interests	26	Declare any competing interests of review authors.	Conflicts of Interest: “The authors declare no conflicts of interest.”
Availability of data, code and other materials	27	Report which of the following are publicly available and where they can be found: template data collection forms; data extracted from included studies; data used for all analyses; analytic code; any other materials used in the review.	Data Availability Statement: data included in article and cited published studies; inquiries to corresponding author.

From: Page MJ, McKenzie JE, Bossuyt PM, Boutron I, Hoffmann TC, Mulrow CD, et al. The PRISMA 2020 statement: an updated guideline for reporting systematic reviews. BMJ 2021;372:n71. doi: 10.1136/bmj.n71.
This work is licensed under CC BY 4.0. To view a copy of this license, visit https://creativecommons.org/licenses/by/4.0/
